# Erratum: Lee et al. Ginseng Extracts, GS-KG9 and GS-E3D, Prevent Blood–Brain Barrier Disruption and Thereby Inhibit Apoptotic Cell Death of Hippocampal Neurons in Streptozotocin-Induced Diabetic Rats. *Nutrients* 2020, *12*, 2383

**DOI:** 10.3390/nu13051656

**Published:** 2021-05-13

**Authors:** Jee Youn Lee, Chan Sol Park, Hae Young Choi, Sung Hyun Chung, Mi Kyung Pyo, Tae Young Yune

**Affiliations:** 1 Age-Related and Brain Diseases Research Center, Kyung Hee University, Seoul 02447, Korea; jeeyoun@khu.ac.kr (J.Y.L.); chansol1028@khu.ac.kr (C.S.P.); neuron@khu.ac.kr (H.Y.C.); 2 Department of Biomedical Science, Kyung Hee University, Seoul 02447, Korea; 3 Department of Pharmacology, College of Pharmacy, Kyung Hee University, Seoul 02447, Korea; suchung@khu.ac.kr; 4 International Ginseng and Herb Research Institute, Geumsan 32724, Korea; pmk67@ginherb.re.kr; 5 Department of Biochemistry and Molecular Biology, School of Medicine, Kyung Hee University, Seoul 02447, Korea; 6 KHU-KIST Department of Converging Science and Technology, Kyung Hee University, Seoul 02447, Korea

The authors have requested that the following changes be made to their paper [1].

The original authors wish to add Dr. Sung Hyun Chung and Dr. Mi Kyung Pyo as a coauthor to this paper. The author contributions were: S.H.C. and M.K.P. contributed to funding acquisition and production of GS-KG9 and GS-E3D, respectively. The updated “Author Contributions” and “Funding” are provided below.

We would like to apologize for any inconvenience caused to the authors and readers by this mistake. The published version will be updated on the article webpage, with a reference to this notice.
